# Predictors of Venous Thromboembolism Following Geriatric Distal Femur Fracture Fixation: Are These Patients at Higher Risk Compared With Hip Fracture Patients?

**DOI:** 10.5435/JAAOSGlobal-D-24-00246

**Published:** 2025-01-17

**Authors:** Anthony E. Seddio, Rajiv S. Vasudevan, Michael J. Gouzoulis, Sahir S. Jabbouri, Jonathan N. Grauer, Brianna R. Fram

**Affiliations:** From the Department of Orthopaedics and Rehabilitation, Yale School of Medicine, New Haven, CT.

## Abstract

**Introduction:**

Venous thromboembolism (VTE) following injury and subsequent fixation of a distal femur fracture (DFFx) is associated with considerable morbidity. However, the incidence of VTE, associated factors, and the relative risk compared with hip fracture (HFx) fixation remains poorly characterized.

**Methods:**

Retrospective cohort study using the PearlDiver M165 database to identify geriatric patients who underwent DFFx and HFx fixation. Clinical risk factors of VTE within 90 days of DFFx and the risk modification associated with enoxaparin (Lovenox) and direct oral anticoagulants (DOACs) relative to aspirin/nonprescription agents were characterized. To determine the odds of VTE following fixation of DFFx relative to HFx, a matched comparison based on age, sex, and Elixhauser Comorbidity Index was done.

**Results:**

Of 24,358 DFFx patients, 1684 (6.9%) developed VTE. Independent risk factors included a prior VTE (odds ratio [OR] 28.76), displaced DFFx morphologies (condylar [OR 5.44], and supracondylar without intracondylar extension [OR 3.96] and with extension [OR 3.75]), active cancer (OR 2.11), coagulopathy disorder (OR 1.15), and younger age (OR 1.03) (*P* < 0.05 for all). Lovenox and DOAC were both associated with reduced odds of VTE (OR 0.40 and OR 0.61, respectively) (*P* < 0.05 for both). Relative to HFx patients, DFFx patients demonstrated heightened odds of VTE (OR 1.25) (*P* < 0.001).

**Discussion:**

This study identified a relatively high rate of VTE, 6.9% within 90 days, following surgical management of DFFx and heightened odds of VTE relative to HFx patients. Various factors demonstrated a notable association with increased odds of VTE, although both Lovenox and DOACs may be effective therapeutic options for risk mitigation.

Distal femur fractures (DFFx) commonly occur as low-energy fragility fractures among geriatric patients, with half of all DFFx occurring in patients ages 70 years and older.^1^ These fractures are associated with morbidity sufficient that 1-year mortality has been reported to range from 13% to 25%.^2-4^ Similar to the management of geriatric hip fractures (HFx), it is critically important to perioperatively optimize this highly comorbid elderly population,^5-8^ to reduce the risk of adverse postoperative outcomes.

One such adverse outcome is venous thromboembolism (VTE), encompassing deep vein thrombosis (DVT) and/or pulmonary embolism (PE).^9^ The classically described risk factors for VTE,^10^ compromising Virchow triad, are vascular endothelial injury, venous stasis, and hypercoagulability.^11^ Although immobilization in the setting of fracture often contributes to the development of VTE,^12^ the regional anatomy of the distal femur may have a unique role in elevating VTE risk due to vascular injury of the adjacent femoral and popliteus veins.^13,14^ DFFx tend to displace apex posteriorly^15^ because of the pull of the gastrocnemius (Figure 1, A). Fracture fragments often cause kinking of posterior vessels seen on preoperative imaging during CT angiogram and therefore may traumatize the adjacent veins (Figure 1, B).

**Figure 1:**
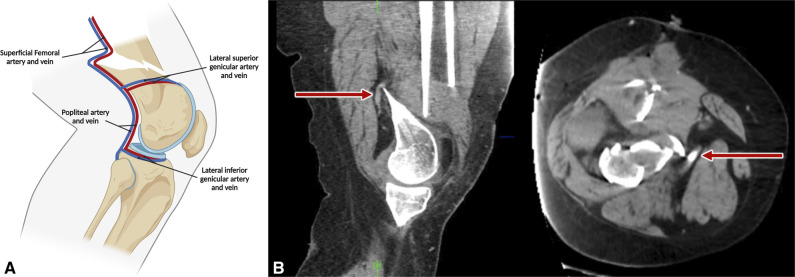
**A**, Lateral illustration of the knee showing vascular proximity of the posterior vessels in a displaced distal femur fracture. **B**, Injury CT angiogram in a patient showing kinking of the popliteus artery over the apex posterior displaced distal femur fracture fragment, with vessel adjacency to fracture seen on sagittal and axial cuts (as shown with the red arrow).

Possibly because of the abovementioned injury-specific anatomy, we anecdotally noted higher rates of VTE at our institution among geriatric low-energy DFFx patients compared with our low-energy HFx population. We found few results in our attempt to corroborate this by published literature review, and of the studies identified, wide variability exists in DFFx VTE rates. For example, in a single-center prospective study by Zhang et al^16^ where all included DFFx patients were screened for DVT,^16^ the rate of VTE following DFFx fixation was 35%, noting that longer surgical duration and increased intraoperative blood loss were associated with even higher risk. Conversely, Onizuka et al^17^ reported a 2.4% VTE rate (1% DVT and 1.4% PE) in their study of geriatric DFFx within 30 days of surgery.

This variability in VTE incidence and paucity of DFFx-specific recommendations for care pathway optimization prompted this study, which leveraged a large, national, multi-insurance administrative data set. Our aim was to identify the rate and risk factors for VTE following geriatric DFFx fixation and characterize the risk modification associated with commonly used thromboprophylaxis agents. In addition, our goal was to evaluate the rate of VTE following surgical fixation of DFFx relative to HFx, a relatively higher frequency fracture and extensively studied population.^18,19^

## Methods

### Data Source and Study Population

This study used data from the 2010—Q3 2022 PearlDiver Mariner 165 database (PearlDiver Technologies). This is a large, national, multi-insurance, administrative claims database, which contains more than 165 million patient records. The use of this database has been established well in the orthopaedics literature.^20-24^ As data from this database are deidentified, our institutional review board has determined studies using this database exempt from review.

**Figure 2:**
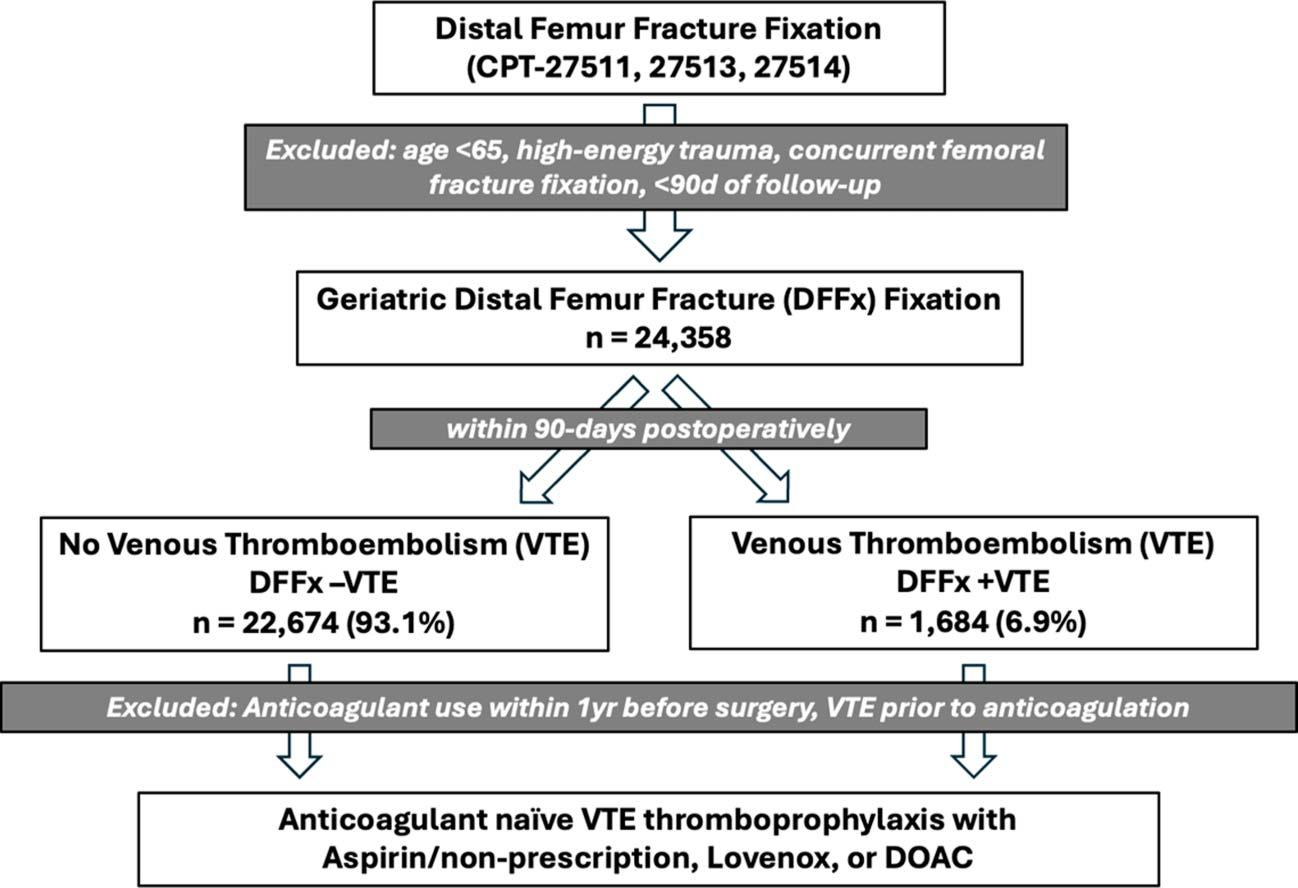
Flow diagram of patient inclusion, exclusions, and assessments.

The flowchart for the identification of patients for the subsequent parts of the study is shown (Figure 2). Patients undergoing first-time fixation of DFFx were identified based on the Current Procedural Terminology (CPT) codes 27511, 27513, and 27514. Exclusion criteria included the following: patients younger than 65 years, those who sustained high-energy mechanisms of injury (eg, motor vehicle collision), those who underwent any other type of femoral fracture fixation on the same day, those with prior ipsilateral total knee arthroplasty, those who were treated nonsurgically, or did not have a minimum of 90 days of follow-up.

To contextualize the rate of VTE following DFFx fixation, patients undergoing geriatric HFx fixation were identified using CPT-27244, CPT-27245, and CPT-27236. The same exclusions as applied to DFFx patients were applied to these HFx fixation patients to improve cohort comparability.

### Patient Management and 90-Day Postoperative Venous Thromboembolism

Annual trend data of DFFx fixation was reported for years with full representation within the data set (2010 to 2021) and normalized per 1,000,000 covered lives. The 90-day postoperative incidence of VTE was determined. This vascular adverse event was defined as the incidence of either DVT and/or PE and identified by International Classification of Disease (ICD) codes appearing within 90 days postoperatively. Patient factors of those with and without VTE following DFFx fixation were then abstracted including age, sex, diabetes, tobacco use, obesity, history of VTE, active cancer, coagulopathy disorder, and prior myocardial infarction (MI). Any adverse event identified in <11 patients was reported as such to protect patient privacy.

Specific DFFx morphology were identified by ICD codes. Three fracture morphologies were assessed: condylar, supracondylar without intracondylar extension, and supracondylar with intracondylar extension. These three fracture types were further subclassified as displaced vs nondisplaced by their unique ICD codes. Patients with unspecified fracture morphology were not assessed by this DFFx morphology subanalysis.

Postdischarge thromboprophylaxis agents were then categorized into exclusive cohorts. These were defined to be enoxaparin (Lovenox), direct oral anticoagulants (DOACs, including apixaban [Eliquis], rivaroxaban [Xarelto], and dabigatran [Pradaxa]), and aspirin/nonprescription. As aspirin can be either prescribed or obtained over the counter, patients with an aspirin prescription or were not prescribed one of the abovementioned agents were included in the aspirin/nonprescription cohort. All medication prescriptions were identified based on prescription billing records using the National Drug Code associated with each agent used within 35 days postoperatively, in accordance with clinical guidelines.^25^ Other agents used for thromboprophylaxis such as fondaparinux and warfarin were excluded due to low postoperative utilization among this geriatric DFFx fixation cohort.

To identify patients using these agents strictly for VTE prophylaxis, we excluded patients from this subanalysis who were prescribed one of these agents as treatment for a postoperative VTE and any patient with an active anticoagulation prescription within 1 year before surgery. Therefore, this analysis involved exclusive cohorts of anticoagulant-naive patients who used these agents as thromboprophylaxis following DFFx fixation, consistent with previously reported methodology.^26^

### Rate of Venous Thromboembolism Following Distal Femur Fracture Versus Hip Fracture Fixation

To compare VTE occurrence following DFFx fixation relative to HFx fixation, these patients were matched 1:4 using an exact match methodology^27^ within the PearlDiver Bellwether software based on age, sex, and Elixhauser Comorbidity Index (ECI) to account for potential differences in underlying patient demographics and comorbidity burden. The incidence and odds of 90-day postoperative VTE were compared between matched patient cohorts of geriatric HFx and DFFx fixation by univariable and multivariable analyses.

### Statistical Analysis

Annual trend data were analyzed using a Z-test for two proportions to compare the incidence of DFFx fixation among geriatric patients between the first year and last year of the study. Univariable analysis of patient characteristics between those who developed VTE and those who did not were compared using independent two-tailed Student *t*-test or Pearson χ^2^ test where appropriate.

Multivariable logistic regression was done to determine the odds ratios (ORs) and associated 95% confidence intervals (95% CIs) of patient factors for those who developed a postoperative VTE relative to those who did not. A second multivariable analysis of the odds of VTE following displaced relative to nondisplaced DFFx morphology was done controlling for age, sex, and ECI. A third multivariable analysis of the odds of VTE following DFFx fixation for patients who exclusively used Lovenox or DOAC relative to aspirin/nonprescription was done controlling for age, sex, and ECI.

All statistical tests were done using the RStudio package embedded within the PearlDiver Bellwether software, with a *P* value of <0.05 considered significant. Tables were constructed using Microsoft Excel (Microsoft Corporation), figures were created with Biorender.com and Prism 10 (GraphPad Software).

## Results

### Patient Management and 90-Day Postoperative Venous Thromboembolism

**Figure 3:**
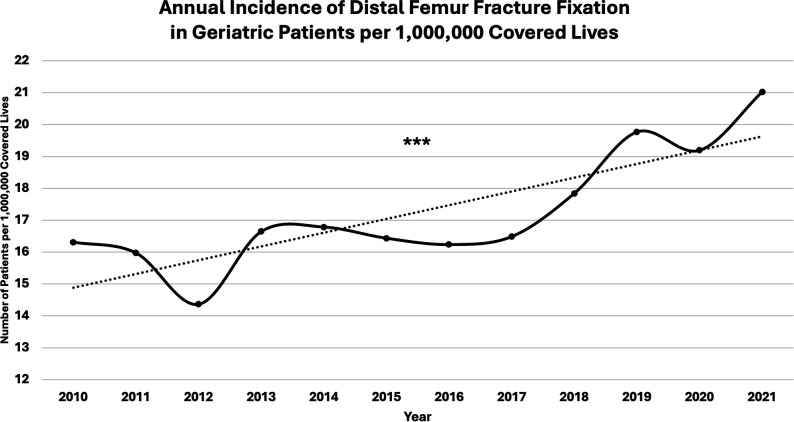
Chart showing annual incidence of distal femur fracture fixation in geriatric patients per 1,000,000 covered lives in the data set from 2010 to 2021. Asterisks represents a statistically significant difference between the first and last data points (*P* < 0.001).

A total of 24,358 geriatric patients were identified who underwent DFFx fixation. Annual incidence of DFFx fixation is shown (Figure 3). Incidence of DFFx fixation per 1,000,000 covered lives in the data set increased by 28.9% between the first year and last year of the study (*P* < 0.001).

**Figure 4:**
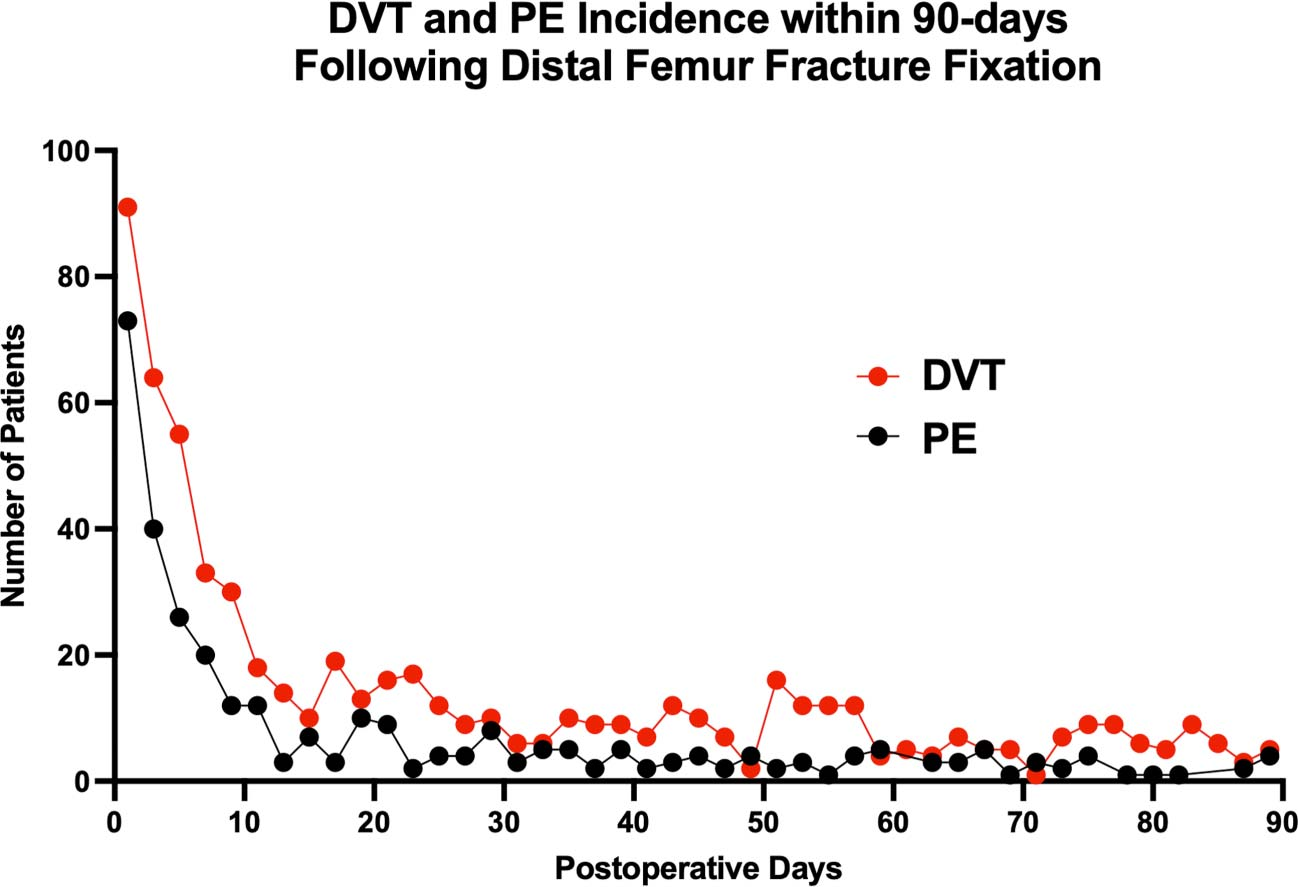
Chart showing incidence of deep vein thrombosis (DVT) and pulmonary embolism (PE) in the 90 days following distal femur fracture fixation.

Of these DFFx fixation patients, 1,684 (6.9%) were diagnosed with a VTE within 90 days postoperatively (DFFx + VTE), whereas 22,674 (93.1%) were not (DFFx − VTE) (Table 1). Most DVTs (698, 54.5%) and PEs (383, 64.9%) occurred within 21 days following surgery (Figure 4).

**Table 1:** Patient Predictors of Venous Thromboembolism Within 90 Days of Geriatric Distal Femur Fracture Fixation

Factor or Variable	Univariable Analysis			Multivariable Analysis	
	DFFx − VTE, N = 22,674 (93.1%)	DFFx + VTE, N = 1684 (6.9%)	*P*	Odds Ratio (95% CI)	*P*
Age ± SD (per year decrease)	74.7 ± 4.5	74.5 ± 4.4	0.170	1.03 (1.02-1.05)	**<0.001**
Sex	—	—	0.125	—	—
Female	19,020 (83.9%)	1388 (82.4%)		Ref	—
Male	3654 (16.1%)	296 (17.6%)		1.04 (0.89-1.21)	0.590
Specific comorbidities	—	—	—	—	—
Diabetes	12,103 (53.4%)	980 (58.2%)	**<0.001**	0.99 (0.88-1.12)	0.893
Tobacco use	7120 (31.4%)	570 (33.8%)	**0.040**	0.90 (0.79-1.01)	0.078
Obesity	8182 (36.1%)	727 (43.2%)	**<0.001**	0.92 (0.82-1.04)	0.183
Clotting risk factors	—	—	—	—	—
Prior VTE	1927 (8.5%)	1217 (72.3%)	**<0.001**	28.76 (25.51-32.47)	**<0.001**
Active cancer	72 (0.3%)	17 (1.0%)	**<0.001**	2.11 (1.07-4.06)	**0.028**
Coagulopathy disorder	4387 (19.3%)	531 (31.5%)	**<0.001**	1.15 (1.01-1.31)	**0.029**
Prior MI	1440 (6.4%)	161 (9.6%)	**<0.001**	0.92 (0.75-1.12)	0.424

CI = confidence interval, DFFx = distal femur fracture, MI = myocardial infarction, OR = odds ratio, VTE = venous thromboembolism

Bold indicates significance *P* < 0.05.

Those who developed postoperative VTE had a higher prevalence of diabetes (*P* < 0.001), tobacco use (*P* = 0.040), obesity (*P* < 0.001), prior VTE (*P* < 0.001), active cancer (*P* < 0.001), coagulopathy disorder (*P* < 0.001), and prior MI (*P* < 0.001) on univariable analysis. Conversely, no differences in age or sex were found on univariable analysis (*P* > 0.05) (Table 1).

On multivariable analysis, the independent patient predictors of postoperative VTE were younger age within the geriatric range (OR 1.03, *P* < 0.001), prior VTE (OR 28.76, *P* < 0.001), active cancer (OR 2.11, *P* = 0.028), and coagulopathy disorder (OR 1.15, *P* = 0.029). By contrast, no notable associations were found with sex, diabetes, tobacco use, obesity, or prior MI (*P* > 0.05) (Table 1).

The most commonly identified DFFx morphology overall was supracondylar fracture without intracondylar extension (Table 2). On multivariable analysis, relative to their nondisplaced counterparts, displaced fractures were consistently associated with greater risk of postoperative VTE: displaced condylar fractures (OR 5.44, *P* < 0.001), displaced supracondylar fracture without intracondylar extension (OR 3.96, *P* < 0.001), and displaced supracondylar fracture with extension (OR 3.75, *P* = 0.009).

**Table 2:** Fracture Morphology Predictors of Venous Thromboembolism Within 90 Days of Geriatric Distal Femur Fracture Fixation

Factor or Variable	Univariable Analysis			Multivariable Analysis	
	DFFx − VTE (n = 22,674)	DFFx + VTE (n = 1684)	*P*	Odds Ratio (95% CI)	*P*
Condylar	—	—	—	—	—
Displaced	850 (3.7%)	141 (8.4%)	**<0.001**	5.44 (3.02-11.04)	**<0.001**
Nondisplaced	141 (0.6%)	18 (1.1%)	0.041	Ref	—
Supracondylar (without intracondylar extension)	—	—	—	—	—
Displaced	1,934 (8.5%)	407 (24.2%)	**<0.001**	3.96 (2.03-9.28)	**<0.001**
Nondisplaced	100 (0.4%)	16 (1.0%)	**0.006**	Ref	—
Supracondylar (with intracondylar extension)	—	—	—	—	—
Displaced	755 (3.3%)	184 (10.9%)	**<0.001**	3.75 (1.58-12.21)	**0.009**
Nondisplaced	49 (0.2%)	<11 (<0.7%)	1.000	Ref	—

CI = confidence interval, DFFx = distal femur fracture, OR = odds ratio, VTE = venous thromboembolism

Multivariable analyses controlled for age, sex, and Elixhauser Comorbidity Index (ECI).

Bold indicates significance *P* < 0.05.

Of patients meeting inclusion criteria for postdischarge thromboprophylaxis analysis (Table 3), exclusive utilization of Lovenox was identified for 1,185 (5.3%), DOAC for 502 (2.3%), and aspirin/nonprescription for 20,596 (92.4%). On multivariable analysis, relative to aspirin/nonprescription patients, prophylaxis with Lovenox, and DOAC revealed lower odds of VTE (OR 0.40 and OR 0.61, respectively) and DVT (OR 0.43 and OR 0.58, respectively) (*P* < 0.05 for all). Conversely, patients prescribed Lovenox revealed lower odds of PE (OR 0.42, *P* < 0.05), whereas patients prescribed DOAC did not (*P* = 0.356).

**Table 3:** Multivariable Analysis of 90-Day Venous Thromboembolism Following Geriatric Distal Femur Fracture Fixation

Factor or Variable	Aspirin/Nonprescription (Reference)	Lovenox	DOAC				
N (%)	20,596 (92.4)	1,185 (5.3)	502 (2.3)				

Factor or Variable	N (%)	N (%)	OR (95% CI)	*P*	N (%)	OR (95% CI)	*P*
VTE	1,292 (6.3)	31 (2.6)	0.40 (0.27-0.56)	**<0.001**	20 (4.0)	0.61 (0.38-0.94)	**0.035**
DVT	1,041 (5.1)	26 (2.2)	0.43 (0.28-0.62)	**<0.001**	15 (3.0)	0.58 (0.33-0.95)	**0.042**
PE	459 (2.2)	11 (0.9)	0.42 (0.22-0.72)	**0.004**	<11 (<2.2)	0.72 (0.32-1.36)	0.356

CI = confidence interval, DOAC = direct oral anticoagulant, DVT = deep vein thrombosis, OR = odds ratio, PE = pulmonary embolism, VTE = venous thromboembolism

Multivariable analyses controlled for age, sex, and Elixhauser Comorbidity Index (ECI).

Bold indicates significance *P* < 0.05.

### Rate of Venous Thromboembolism Following Distal Femur Fracture Versus Hip Fracture Fixation

After matching, there were 24,347 DFFx and 97,384 HFx patients who had undergone surgical fracture fixation. The incidence of 90-day postoperative VTE for these matched cohorts was 6.8% vs. 5.6% (for DFFx vs. HFx, respectively) (*P* < 0.001). On multivariable analysis of DFFx fixation, relative to HFx fixation, DFFx patients demonstrated higher odds of VTE (OR 1.25, 95% CI, 1.18 to 1.32) (*P* < 0.001).

## Discussion

This study investigated the rate of and risk factors for VTE following low-energy DFFx fixation in geriatric patients. The rate of surgically managed geriatric DFFx from 2010 to 2021 has grown, mirroring the aging U.S. population.^25^ The overall rate of 90-day VTE was found to be 6.9% in these patients. Although both DFFx and HFx contribute to postoperative VTE risk,^16,17,26,28^ our matched cohort analysis found DFFx VTE incidence to be 6.8%, whereas geriatric HFx VTE incidence was 5.6%. This finding, meant to contextualize the relative postoperative VTE risk of these patients, underscores the importance of fracture-specific care pathway optimization for DFFx patients who may be uniquely more vulnerable to VTE.

Prior single-center studies have reported wide variability in VTE incidence following DFFx.^16,17,29^ Among 24,358 geriatric DFFx patients in this study, incidence of 90-day postoperative VTE was identified for 1,684 patients (6.9%), which is lower than the 35% VTE rate previously reported by Zhang et al,^16^ but higher than the 2.4% VTE rate found by Onizuka et al.^17^ This VTE rate variability may be explained in part by the prospective nature of the study by Zhang et al, where patients were screened for DVT, and therefore, asymptomatic VTE would have been captured that might not otherwise have been in routine clinical practice. This differs from this study methodology and the study of Zhang et al, where one would expect only symptomatic patients to be diagnosed. In addition, our study looked at 90-day rates of VTE, whereas Onizuka et al limited analysis to 30 days, potentially explaining their lower VTE rate.

We identified history of VTE (72.3% of VTE patients) as the strongest predictive factor for VTE following DFFx fixation (OR 28.76), which is consistent with the prior literature.^16^ Active cancer at time of DFFx fixation (1.0% of VTE patients) contributed the second greatest risk factor for VTE (OR 2.11), consistent with the established relationship between cancer and a hypercoagulable state.^30-32^ Expectedly, history of coagulopathy disorder (31.5% of VTE patients) was also associated with heightened VTE risk (OR 1.15). Together, these findings emphasize the need for VTE risk stratification within care pathways for DFFx management.

Our study found that a displaced DFFx was associated with greater risk of VTE compared with nondisplaced DFFx. This could in part be explained by an increase in local vascular disruption and endothelial damage of the adjacent deep posterior veins when fracture fragments displace, which is a key element of Virchow triad.^11^ It is also possible that the additional surgical time associated with surgical exposure needed to perform fracture fixation of a displaced DFFx contributes to this heightened VTE risk, which is corroborated by the findings of Zhang et al,^16^ revealing that longer surgical time and blood loss were associated with elevated VTE risk. As displacement and surgical time/blood loss would be expected to correlate, the causality of these findings warrant future prospective analysis.

These relationships between local vascular anatomy, extended surgical time, and blood loss may account for our finding that DFFx patients were at even greater risk of 90-day VTE (OR 1.25) compared with HFx patients on matched analysis. Although likely multifactorial, this heightened risk may be related to weight-bearing status protocol differences between HFx and DFFx. HFx patients are often weight-bearing as tolerated postoperatively,^33,34^ whereas DFFx patients may have greater weight-bearing restrictions,^35^ this may contribute to different levels of venous stasis, another key element of Virchow triad.^11^ Regardless of the cause, our study suggests that DFFx patients have notable VTE risk, which emphasizes the need to refine care pathways for DFFx patients to mitigate this risk.

Chemical VTE thromboprophylaxis has become the standard of care in orthopaedic surgery,^25,36,37^ yet no consensus exists on the optimal prophylactic agent following DFFx fixation. Our study found that most VTEs occurred in the first 21 days after surgical fixation of DFFx, highlighting this early postoperative window as a critically important period for VTE risk reduction. This time frame of heightened postoperative VTE risk is consistent with both prior orthopaedic literature^16,17^ and general trauma literature.^38,39^

Our study suggests superior efficacy of both Lovenox and DOACs relative to aspirin/nonprescription in the prevention of postoperative VTE following DFFx fixation. The association between VTE risk reduction in patients prescribed DOACs in this setting adds to the increasing body of evidence supporting their efficacy and safety following orthopaedic surgery.^40^ These DOAC agents may be a particularly favorable therapeutic strategy due to their oral route of administration^41^ and predictable pharmacokinetics, which do not require routine monitoring.^42^ Importantly, our study highlights the potentially greater efficacy of DOACs in the management of DVT relative to PE, as the odds of PE when individually stratified was not markedly modified by DOAC agents. With recent literature highlighting the thromboprophylaxis potential of aspirin as monotherapy,^43^ future studies should aim to prospectively elucidate the association with VTE risk modification for DFFx patients undergoing surgical fixation. These analyses should ensure that utilization of over-the-counter aspirin preparations are considered in addition to prescribed aspirin as was included in this study.

This study has several strengths as a large, nationally representative, and generalizable sample of patients; however, important limitations exist. As with any retrospective database study, the data are reliant on the accuracy of the administrative coding. In addition, the exact dose and precise day within the 35-day postoperative window a patient may have stopped anticoagulation is not captured well due to limitations in prescription drug coding, which should be further explored in future prospective analyses. Another limitation is that our analysis is unable to stratify patients based on implant construct used in the DFFx fixation due to CPT coding limitations, which remains an area for further research. In addition, the used database captures postdischarge prescriptions, but the associated risk reduction for the use of these agents during admission remains an area of future research.

## Conclusion

The current study of geriatric DFFx revealed that various patient factors and displaced fractures of various morphologies were associated with heightened VTE risk. Interestingly, DFFx was associated with heightened VTE risk compared with a matched cohort of HFx patients, which we hypothesize may be related to the local disruption of adjacent veins in DFFx and differences in postoperative weight-bearing protocols. Although consensus regarding the optimal VTE chemical prophylactic agent does not currently exist for DFFx, based on this retrospective national database analysis, Lovenox and DOACs may both be effective options. The considerable risk for postoperative VTE seen in geriatric DFFx patients warrants further analysis, as the observed findings suggest opportunities for streamlining the identification of high-risk patients and optimizing perioperative care pathways.
